# Nanoscopic Characterization of Starch-Based Biofilms Extracted from Ecuadorian Potato (*Solanum tuberosum*) Varieties

**DOI:** 10.3390/polym16131873

**Published:** 2024-06-30

**Authors:** Pablo Ilvis, José Acosta, Mirari Arancibia, Santiago Casado

**Affiliations:** Food and Biotechnology Science and Engineering Department, Technical University of Ambato, Ambato 180207, Ecuador

**Keywords:** starch biofilms, potato starch, atomic force microscopy, nanoscience, biopolymers

## Abstract

Synthetic plastic polymers are causing considerable emerging ecological hazards. Starch-based biofilms are a potential alternative. However, depending on the natural source and extraction method, the properties of starch can vary, affecting the physicochemical characteristics of the corresponding casted films generated from it. These differences might entail morphological changes at the nanoscale, which can be explored by inspecting their surfaces. Potato (*Solanum tuberosum*) is a well-known tuber containing a high amount of starch, but the properties of the biofilms extracted from it are dependent on the specific variety. In this research, four Ecuadorian potato varieties (*Leona Blanca*, *Única*, *Chola*, and *Santa Rosa*) were analyzed and blended with different glycerol concentrations. The amylose content of each extracted starch was estimated, and biofilms obtained were characterized at both macroscopic and nanoscopic levels. Macroscopic tests were conducted to evaluate their elastic properties, visible optical absorption, water vapor permeability, moisture content, and solubility. It was observed that as the glycerol percentage increased, both moisture content and soluble matter increased, while tensile strength decreased, especially in the case of the *Chola* variety. These results were correlated to a surface analysis using atomic force microscopy, providing a possible explanation based on the topography and phase contrast observations made at the nanoscale.

## 1. Introduction

Environmental pollution caused by synthetic plastics is considered poorly reversible [1], affecting many ecological areas. Weathering of these plastics is emerging as a global problem [2,3], mainly because of their biodegradability and the easy dispersion of the resulting microplastics [4]. It has been proved that microplastics are currently present in animals and plants [5,6], affecting their metabolism [7,8].

Synthetic plastic emissions should be stopped, but human requirements are increasing at a rate that exceeds its mitigation [9]. Substitution by more biodegradable polymers is crucial, but the physicochemical properties of these bioplastics may not meet all the requirements. It is necessary to explore sustainable, easy, and cheap procedures to generate optimal biofilms with appropriate characteristics.

Biofilms generated from starch polymerization constitute a promising alternative. Starch is present in many tubers and grains, and its extraction is straightforward. However, the properties of its casted films are quite dependent on the starch composition [10,11,12,13]. Hence, the search for the optimal starch-based biofilm with industrial interest entails exploring the starch source, too. Andean tubers have great potential [14], and some of us have already examined the properties of films extracted from a variety of them [15]. It was found in this article ([15]) that the film with the highest elastic modulus (Young’s modulus) is the one formed from starch extracted from potato, in comparison to others cast from starch from other tubers. Nevertheless, there are plenty of different Andean potato species, and the resulting elastic properties of films formed from starch extracted from each of them differ [13,14,15,16,17,18].

Here, we inspect the properties of biofilms generated from four different varieties of Ecuadorian potatoes (*Solanum tuberosum*): *Leona Blanca*, *Única, Chola*, and *Santa Rosa*, blended with varying concentrations of glycerol as a plasticizer. Initial amylose content and resulting physicochemical macroscopic properties such as tensile strength, Fourier Transform Infrared (FTIR) absorption, visible optical absorption, water vapor permeability, moisture content, and solubility are measured. Optical and atomic force microscopies are analyzed, to characterize the nanoscopic structure of each biofilm surface, finding that there may exist a correlation between the macroscopic properties and the surface corrugation at the nanoscale. This analysis would provide information about the characteristics of films gathered from starch extracted from the species *Leona Blanca*, *Única*, *Chola*, and *Santa Rosa*, which would permit determining the most appropriate variety depending on the biofilm application purpose.

## 2. Materials and Methods

### 2.1. Starch Extraction

Starch in this research was extracted from four potato (*Solanum tuberosum*) varieties, denoted as *Leona Blanca*, *Única*, *Chola*, and *Santa Rosa,* using the methodology described by Pico et al. [15]. All the tubers were peeled, washed, cut into small pieces, and grounded. Distilled water was added to this extract until a volume of 1000 mL was reached. Solutions were filtered using a gauze filter with a pore diameter of approximately 120 mesh. They were allowed to settle in a precipitation vessel for 6 h. Afterward, the supernatant was discarded, and the starch powder was placed in an oven at 45 ± 5 °C overnight. The weight proportion of starch extracted from raw potatoes was around 14% in all cases studied.

### 2.2. Determination of Amylose Content

To correlate the results to the amylose content of each variety, a colorimetric method similar to that described by Bates et al. [19] was used. Solutions of 1% *w*/*v* (g/mL) potato starch, 1% *w*/*v* potassium iodide (KI) solution, and 0.2% *w*/*v* iodine solution (I:KI) were prepared. Since iodine is not soluble in water, this solution was dissolved with 10% *w*/*v* KI, as was performed by Bahdanovich et al. [20]. 0.5 mL of the 1% *w*/*v* potato starch solution was added to a precipitation vessel, followed by the addition of 2 mL of the 1% *w*/*v* potassium iodide solution. The mixture was then stirred and allowed to stand for 10 min. 2 mL of the 0.2% *w*/*v* iodine solution was added to the mixture. In parallel, a blank sample was prepared using the same solutions, where 0.5 mL of distilled water was used instead of the sample. The absorbance of the solutions was measured at 600 nm using an accuSkan GO UV-Vis spectrophotometer (Thermo Fisher Scientific, Waltham, MA, USA). Measurements were performed in triplicate. An estimation of the amylose content relative to each variety could be obtained using Equation (1) from Duan et al. [21].

(1) $$\%\;Amylose=\frac{{Abs}_{600}}{Correction\;factor}\times 100$$

*Abs* represents the average absorbance readings dependent on the variety, and the *Correction factor* is 1.3, according to Lu et al. [22].

### 2.3. Film Preparation

Starch biofilms of each potato variety were prepared by mixing 7 g of potato starch with 100 mL of distilled water. Solutions were heated up to a temperature of 90 °C using a hotplate and a magnetic stirrer at a constant rate of 200 rpm. Different amounts of glycerol (0, 1, 2, 3, and 4 g) were introduced into the mixture, following the methodology outlined by Farhan and Hani [23]. Once the solution appeared homogeneous, 20 mL portions were poured into 100 × 10 mm Petri dishes. Subsequently, these Petri dishes were placed in an incubator set at 45 °C for 24 h. Finally, generated films were removed from the Petri dishes and stored in desiccators under ambient conditions.

### 2.4. Optical Characterization

The visual appearance and apparent transparency of each biofilm sample were obtained using a conventional camera and diffused white light, placing the films onto a background containing black letters and figures.

Optical micrographs were measured by fixing square sections of an approximately 10 mm side from each starch-based film under an EVOS XL microscope (Life Technologies, Thermo Fisher Scientific). Each sample was attached to a microscope slide using adhesive tape, positioned inverted, irradiated with transmitted white light, and inspected using a 40× magnification and 0.65 numerical aperture (in air) collimated objective. Similar optical micrographs were obtained using a reflected white light microscope (Olympus Corporation of the Americas, Center Valley, PA, USA).

### 2.5. Mechanical Properties

Tensile strength, elastic modulus, and elongation at the break of the biofilms were determined under ambient conditions using a modified version of the ASTM D 882-88 standard method with a Brookfield CT3 texture analyzer (Ametek, Berwyn, PA, USA). Biofilms were cut into rectangular strips measuring 50 mm in length and 20 mm in width. The separation speed between the crosshead was set at 0.5 mm/s, and the results were averaged from 5 replicates of each sample.

### 2.6. Fourier Transform Infrared Spectroscopy (FTIR)

Fourier transform infrared (FTIR) spectra were recorded according to the method described by Orsuwan et al. [24] using a Spectrum Two spectrophotometer (Perkin-Elmer, Waltham, MA, USA) coupled to an attenuated total reflectance (ATR) adapter. All measurements covered a wave number range between 4000 and 600 cm^−1^ with 4 cm^−1^ accuracy. Under ambient conditions, samples of each biofilm were located directly on the ATR tip surface and gently pressed with the flat-tip plunger. Data were acquired in triplicate at different locations of each film.

### 2.7. Visible Optical Absorption

To measure the opacity of the biofilms, an accuSkan GO UV-Vis spectrophotometer (Thermo Fisher Scientific, Waltham, MA, USA) was employed. Samples were cut into rectangular pieces commensurate with the internal size of the quartz cells fitting the equipment. Measurements were taken in triplicate under ambient conditions, covering a wavelength range from 200 nm to 600 nm. Opacity (*O*) was calculated using Equation (2). Film thickness (*X*, mm) was measured using a 0.01 mm accuracy micrometer caliper, averaging the data from eight different locations on each film. Absorption was measured at 560 nm (*Abs*_560_).

(2) $$O=\frac{{Abs}_{560}}{X}$$

### 2.8. Water Vapor Permeability (WVP)

For water vapor permeability (*WVP*) determination, a modification of the method proposed by Sobral et al. [25], outlined in Pico et al. [15], was employed. Glass chambers with a circular opening of 33 mm in diameter were used. An equal amount of dry silica gel (previously dried at 105 °C) was placed inside each chamber, and the entrance was completely sealed with each of the formed bioplastic films. Whole chambers were exposed to saturated water vapor under ambient conditions for 10 h and weighted at hourly intervals. All measurements were conducted in triplicate, and the water vapor permeability value was determined using Equation (3).

(3) $$WVP=\frac{w\;x}{t\;A\;∆P}$$

Relation of the weight *w* against time *t* was obtained by fitting experimental data to a linear regression. Thickness *x* was measured using a 0.01 mm accuracy micrometer caliper, averaging the data from eight different locations on each biofilm. The permeation area *A* corresponds to the opening of each glass chamber. ∆*P* represents the difference in partial vapor pressure between the atmosphere inside the silica gel and pure water (2642 Pa at 22 °C).

### 2.9. Moisture Content (MC)

For this test, a gravimetric method was applied using a conventional precision scale (0.01 mg accuracy). Samples of similar sizes were weighed without undergoing any prior treatment, and values were recorded as the initial weight (*W*_0_). Subsequently, they were subjected to a drying process in an oven at 105 °C for 24 h. After the specified time, they were removed from the oven and placed into a desiccator with silica gel until they cooled down. Once acclimatized, they were weighed again, and values were recorded as the final weight (*W_f_*). All measurements were performed in triplicate. The moisture content (*MC*) was calculated using Equation (4).

(4) $$MC=\frac{W_{0}-W_{f}}{W_{0}}\times 100$$

### 2.10. Water Total Soluble Matter (TSM)

Water solubility (Total Soluble Matter, *TSM*) was measured following the method described by Arancibia et al. [26] and Salazar et al. [27] using a conventional precision scale (0.01 mg accuracy). Square samples of approximately 5 mm sides from each biofilm were weighed, and their initial weights (*W_i_*) were obtained. Samples were then submerged into bottles containing 30 mL of distilled water, which were placed on a shaker operating at 70 rpm for 24 h at room temperature. After retrieving the samples, they were stored at 105 °C for 24 h. Subsequently, the final dry weight (*W_f_*) of each sample was determined, and their content of *TSM* was calculated using Equation (5).

(5) $$TSM=\frac{W_{i}-W_{f}}{W_{i}}\times 100$$

### 2.11. Atomic Force Microscopy (AFM) Characterization

To characterize the surface of each starch biofilm at the nanoscale, a Park System XE7 Atomic Force Microscope (AFM, Santa Clara, CA, USA) was employed. For this analysis, a small sample from each biofilm was cut and placed onto the magnetic disk (sample holder) of the AFM employing a double-sided tape. Samples were scanned in tapping mode under ambient conditions using PPP CONTSCR cantilevers (0.2 N/m, 23 kHz, <10 nm in diameter, nominal values). The resolution was set at 512 × 512 pixels^2^. Images were processed using the XEI 5.1.6.Build1 software (Park System, Santa Clara, CA, USA), applying a polynomial background subtraction. The AFM has a proven capacity to measure the surface of very soft materials like living cells [28] and to relate nano- and microstructures to macroscopic properties resulting from them [29] without requiring metal coating or vacuum on the sample. Hence, this technique offers the possibility of directly determining the nanostructure of biofilm samples without significantly perturbing them.

### 2.12. Statistical Analysis

The statistical analysis was conducted by determining variance (ANOVA) and Tukey’s multiple comparison tests at a 95% confidence level using the R 4.4.0 software (R Development Core Team, Vienna, Austria).

## 3. Results and Discussion

### 3.1. Amylose Content

Estimations of amylose weight–weight concentrations from the different potato varieties are shown in Table 1. These values align closely with those reported by Zhao et al. [30], which obtained similar percentages of amylose content, ranging from 20% to 26%. Zhao et al. [18] also presented values in the same range: from 19.2% to 26%. Additionally, McGrance et al. [31] reported an amylose content of 29.7% ± 10.3%.

It is important to note that a higher amylose content contributes to greater rigidity and strength in the constructed biomaterial, while a lower amylose content is related to a higher flexibility [32]. This may be caused by a different polymerization process occurring inside the biofilms during the curing process. A higher amylose content might yield a more regular and rigid but fragile structure, while higher amylopectin content enhances the biofilm’s capacity to bend but with reduced elastic resistance.

### 3.2. Optical Characterization of the Biofilms

**Figure 1:**
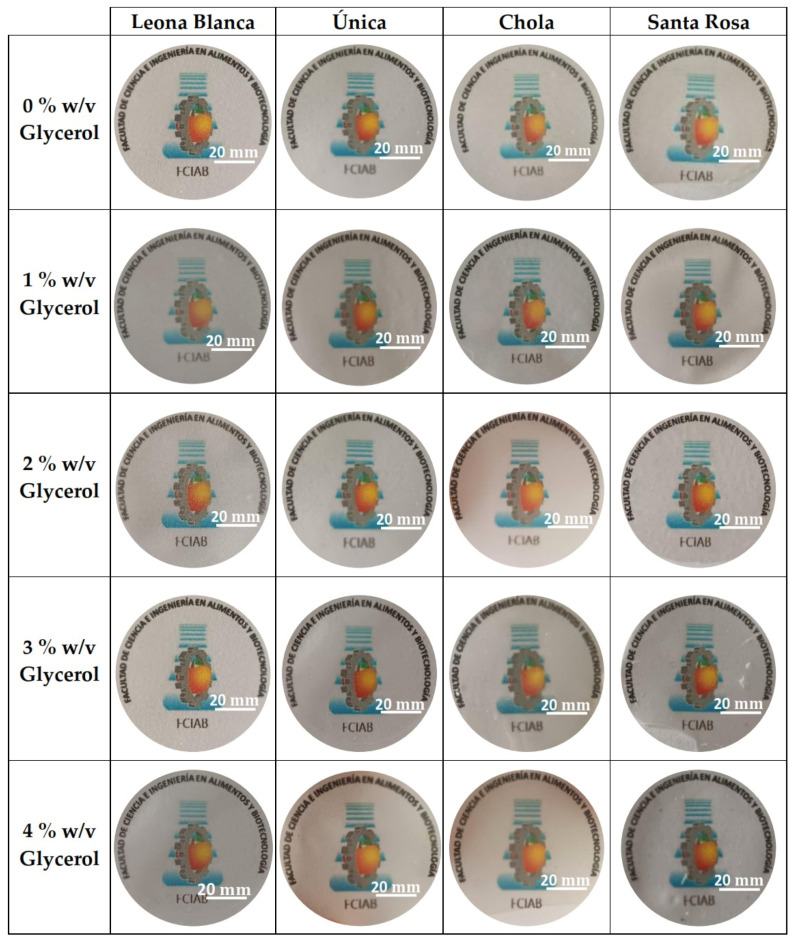
Visual appearance and apparent transparency of biofilms obtained from starch from different potato varieties at different percentages of glycerol.

The visual appearance and apparent transparency of the biofilms are presented in Figure 1. Biofilms generated without glycerol showed more apparent clearness. Those biofilms formed from starch extracted from the *Única* variety without glycerol exhibit good transparency, but they become opaquer when this plasticizer is added. This opacity might be related to the molecules’ entanglement during the curing process. Finally, biofilms extracted from the *Santa Rosa* variety exhibit a yellowish color at low glycerol percentages.

**Figure 2:**
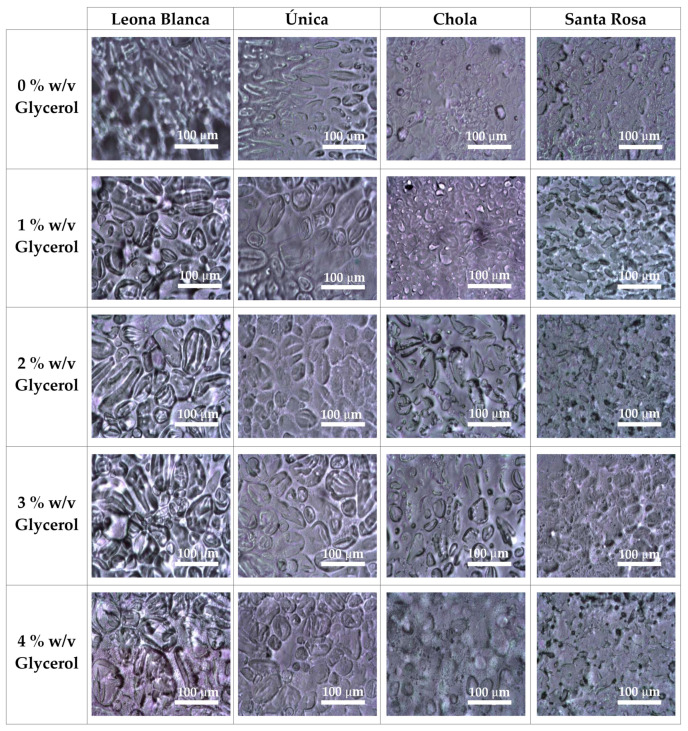
Micrographs of the biofilms obtained from starch from different varieties of starch and glycerol percentages were obtained through the optical white light transmission microscope.

Transmitting white light optical microscopic inspection also revealed differences between the structures of each sample blended with different percentages of glycerol, as observed in Figure 2. Samples acquired from starch from the *Leona Blanca* type presented a significant contrast. On the other hand, those obtained from starch from the *Única* variety display a ramified apparent structure but with lower contrast. Regarding films based on starch extracted from the *Chola* potato variety, a very good interaction is observed, with starch and glycerol seemingly more fused. Finally, inspection of films from starch extracted from *Santa Rosa* type revealed a possible structure change after glycerol addition. Similar results can be observed in reflection white light optical microscopic images (Figure S1).

### 3.3. Elastic Properties

**Figure 3:**
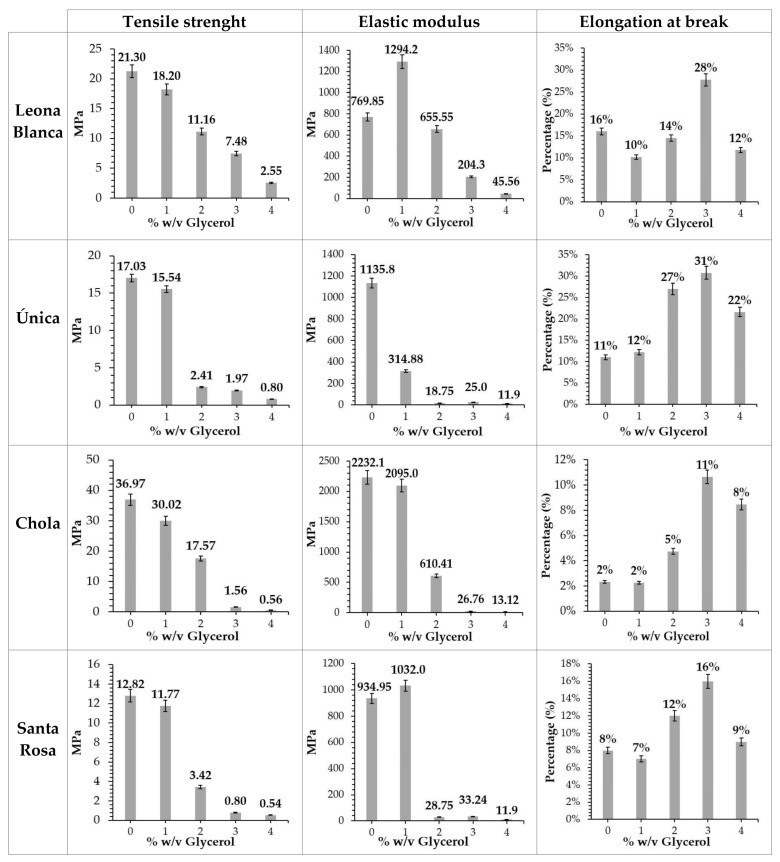
Mechanical properties obtained from the biofilms generated from starch from each potato variety and glycerol percentages analyzed. The data shown (mean ± SD) are representative of five independent experiments.

Figure 3 shows the tensile strength, modulus of elasticity, and elongation at break data obtained on each biofilm produced from each potato variety and described glycerol percentages.

Biofilms prepared without glycerol as a plasticizer exhibited higher tensile strength (36.97, 21.30, 17.03, and 12.82 MPa for *Chola*, *Leona Blanca*, *Única*, and *Santa Rosa*, respectively). Furthermore, it is observed that, in general, the higher the percentage of glycerol added, the lower the tensile strength but the higher the elongation at break (up to a certain limit, after which it diminishes). This phenomenon could be attributed to the presence of intermolecular hydrogen bonds between starch and glycerol, conferring more plasticity but less tensile strength on the films [33,34,35,36].

Films prepared from starch from the *Chola* variety exhibit the highest tensile strength and modulus of elasticity at 1% *w*/*v* glycerol (30.02 and 2095.3 MPa, respectively). These values surpass those reported elsewhere [13,37]. Instead, films formed from starch extracted from *Única* and *Santa Rosa* varieties, at glycerol percentages higher than 1% *w*/*v*, present lower tensile strength values compared to those published in the literature [16].

Differences in mechanical properties of the biofilms analyzed, blended with starch extracted from the potato varieties explored, could be related to the amylose content shown in Table 1. Amylose tends to form stronger biofilms than amylopectin. Hence, an increase in the amylose content may induce tensile strength growth [13].

### 3.4. Fourier Transform Infrared Spectroscopy (FTIR) Absorption

**Figure 4:**
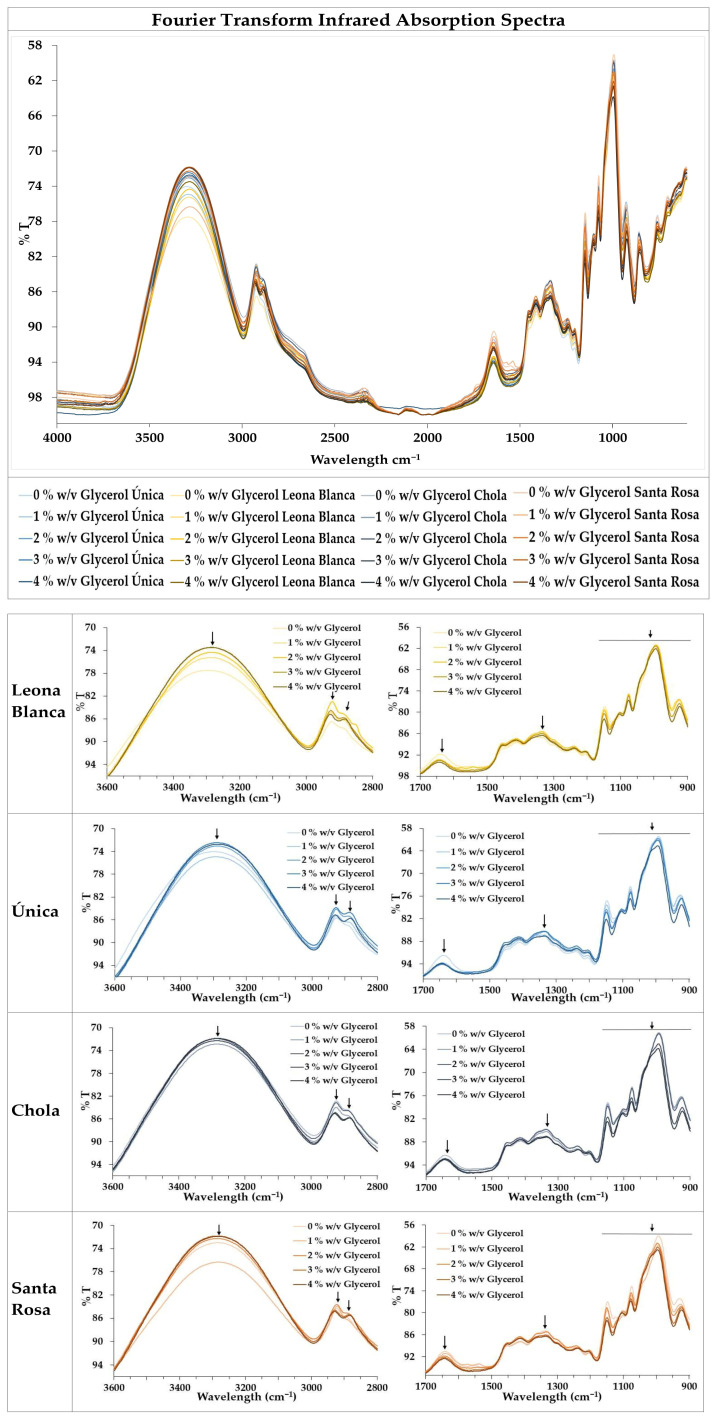
Infrared absorption spectra by Fourier transform were obtained in each of the biofilms produced. Arrows highlight representative peaks.

Fourier-transform infrared (FTIR) spectroscopy allows the observation of characteristic bands corresponding to vibrations of chemical bonds present in each biofilm. Spectrograms recorded reveal a similar conformation of the starch-based biofilms obtained from all the potato varieties used, with slight differences. Results are shown in Figure 4.

The region between 4000 and 3000 cm^−1^ is sensitive to the water content in the sample, indicating the presence of absorbed water in all the potato starch-based biofilms.

Biofilms analyzed also exhibit a dominant broad band appearing at around 3272 cm^−1^. This absorption peak can be related to a stretching vibration occurring at hydroxyl (OH) groups associated with the glycerol and starch molecules present in the biofilms [33,38]. A change in this peak is observed in most of the cases after glycerol addition, suggesting a possible OH interaction between starch and glycerol. Remarkably, the *Chola* potato variety displays very little divergence at this peak upon glycerol addition. This could be related to a better entanglement between starch and glycerol molecules in this variety, in agreement with the mechanical properties recorded.

Bands between 2942 and 2874 cm^−1^ may correspond to stretching vibrations of the CH groups composing the starch [15]. These bands were slightly shifted towards higher wavelengths, reinforcing the indication that the addition of glycerol promotes hydrogen bonding interactions between starch and glycerol [39]. The band around 1638 cm^−1^ can be related to the flexural vibrations of the OH and CH groups [40], the peak found between 1390 and 1380 cm^−1^ can be attributed to the flexural vibration of COH [24], and the bands in the region between 1200 and 900 cm^−1^ may correspond to vibrations of the CO, CC, and COH groups [39]. Some small differences in the band shape and intensity can be observed due to interactions between the glycerol and the starch. In all cases, the band at 1000 cm^−1^ became more prominent in biofilms without glycerol, which could be related to changes in the amorphous or crystalline transitions in the biofilms [41].

### 3.5. Optical Absorption

The thickness of all the biofilms explored in this analysis is revealed in Table 2.

Table 3 shows the opacity values obtained from each potato variety and glycerol percentage, calculated based on the thickness of each biofilm revealed in Table 2. It can be observed that at 1% *w*/*v* glycerol, the biofilm produced from the *Chola* variety exhibits the highest opacity value (1.94 mm^−1^) compared to the other varieties. This value is lower than a previously reported result [37] but higher than others published elsewhere [13]. This phenomenon is probably connected to the different entanglements occurring during the polymerization process mentioned before. Internal conformations inside the films may generate varying amounts of optical diffraction points due to the polymerization of its constituents. A higher opacity value may indicate a denser appearance of tangled structures inside the films. On the other hand, more transparency might imply more regularity. In the *Chola* case, glycerol at low concentrations lowers slightly the mechanical elastic modulus but increases the opacity, suggesting that it could be generating aggregations. Instead, opacity and elastic modulus are reduced providing enough glycerol, but elongation at break is clearly enhanced, suggesting a more flexible and regular binding. If glycerol is too high, plasticity is decreased, and opacity is increased.

### 3.6. Water Vapor Permeability (WVP)

Water vapor permeability (*WVP*) results are outlined in Table 4. The data shown correspond to measurements performed in triplicate and represent the mean and the standard deviation.

Data gathered from biofilms corresponding to the *Leona Blanca* variety blended with 4% *w*/*v* glycerol show higher values compared to those collected from the other varieties explored here but are lower than data previously reported in other studies [15]. It is observed that as the percentage of glycerol increases, *WVP* also increases. *WVP* values below 45 mg mm min^−1^ m^−2^ kPa^−1^ could be attributed to a good interaction between starch and glycerol, which form a compact structure, resulting in lower *WVP* values [33].

### 3.7. Moisture Content (MC)

Moisture content (*MC*) results are outlined in Table 5. The data shown correspond to three replicates and represent the mean and the standard deviation of each case.

It can be noticed that *MC* values of every variety studied increase when the percentage of glycerol increases. However, even without glycerol, there are *MC* differences among the biofilms analyzed. Values of biofilms formed from starch from the *Chola* and *Leona Blanca* varieties are close to those published elsewhere [15,17,42].

### 3.8. Water Total Soluble Matter (TSM)

Total Soluble Matter (*TSM*) in water results are outlined in Table 6. The data shown correspond to the measurements performed with three replicates and represent the mean and the standard deviation.

*TSM* results were found to follow the same trend as the *MC*. Considering both measurements, a higher percentage of glycerol seems to be related to a greater solubility of the generated films, although *TSM* values might be affected by glycerol release, too. Biofilms obtained from starch from *Leona Blanca* have lower values up to 3% *w*/*v* glycerol, but at 4% *w*/*v* glycerol *TSM* is slightly higher than in the *Chola* and *Única* cases. Additionally, it can be observed that biofilms obtained from starch extracted from *Santa Rosa* type are more soluble in water than other varieties, maybe due to the plasticizer interaction.

Potato *TSM* values of 15.64 ± 0.56 [43] and 24.43 ± 0.13 have been published [15]. Based on the results shown in Table 5, the *Chola* and *Leona Blanca* types are the varieties having closer values to those reported in these studies.

### 3.9. Nanoscopic Characterization

**Figure 5:**
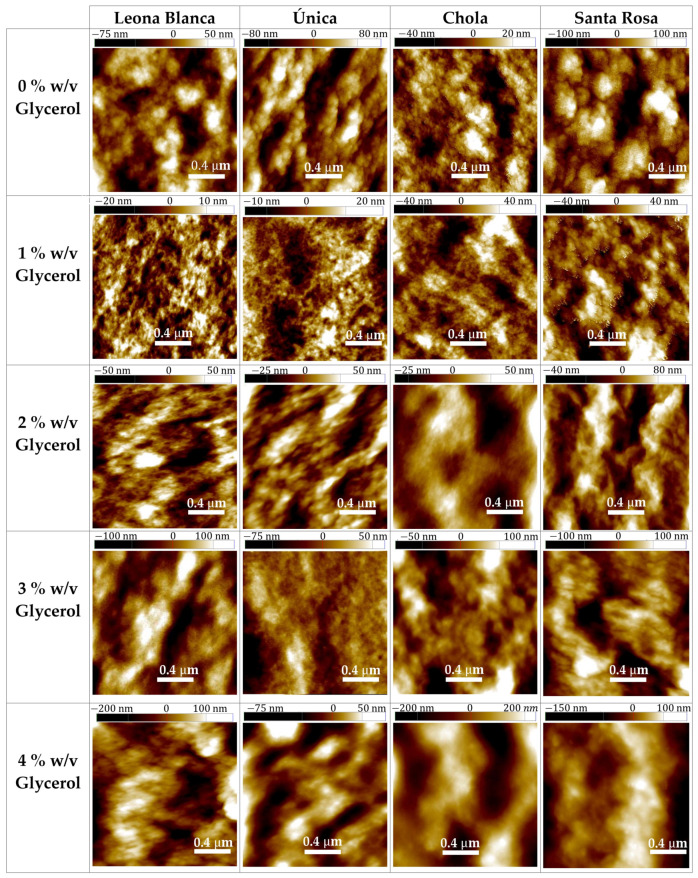
Tapping mode Atomic Force Microscopy topographic images on the surface of biofilms based on starch extracted from each potato variety blended with different glycerol percentages.

Tapping mode Atomic Force Microscopy (AFM) images are shown in Figure 5. A comparison of topographies in various areas of the biofilms formed from starch from each analyzed variety is presented. All biofilms without glycerol exhibited a granular morphology, probably caused by the presence of spherical or oval granules derived from the starch used in their production [13]. With this plasticizer, corrugation changes from a peaky and flat surface at lower concentrations to a smoother but microscopically uneven surface if the amount of glycerol provided to the blends increases.

Differences can be observed comparing the films obtained from starch extracted from distinct potato species. For instance, without glycerol, bigger aggregates can be detected in *Leona Blanca*, *Única*, and *Santa Rosa* cases, but in the *Chola* corresponding film, smaller and scattered beads can be seen at its surface. These beads might act as potential optical diffusion points. If low concentrations (1% *m*/*v*) of glycerol are added, aggregations of the films formed from starch from *Leona Blanca*, *Única*, and *Santa Rosa* species seem to disappear at the surface. A flatter and peaky structure is observed, especially in the cases of *Leona Blanca* and *Única.* In the *Chola* case, corrugation shows a negligible change after glycerol inclusion. Beyond this first step, the surfaces of all samples seem to become smoother at the nanoscale if this plasticizer’s concentration increases, but more irregularities at the microscale appear, showing prominences of flat sides. This might also allow a better understanding of opacity results at high glycerol concentrations, since microscopic height variability could enhance optical diffusion at their surfaces more than if these imperfections were nanoscopic and irregularly distributed. A roughness analysis was performed on each image recorded, which corroborates the observation. The following parameters were calculated: the average roughness between the profile and its mean surface or Ra (Table S1), the arithmetic average of the five highest peaks and five lowest valleys or Rz (Table S2), and the root mean squared roughness or Rq (Table S3). A three-dimensional representation of all the images recorded is also provided to allow a more detailed visualization of the topographic differences (Figure S2).

Phase contrast observed is related to the variation of energy dissipation occurring at each pixel, providing information about the adhesion of the tip or the distinct mechanical properties of the sample’s surface at the nanoscale. Phase contrast observed (Figure S3) among the analyzed biofilms revealed structural differences, more pronounced in the biofilms prepared with 4% *w*/*v* glycerol. Segregations shown could be attributed to a higher capacity to adsorb water molecules onto their surface, which may be related to a change in polarity at the interface [15]. Increasing phase contrasts can be observed upon increasing glycerol content in every species, coinciding with the *MC* data trend. On the other hand, a comparison between phase contrasts among films without glycerol retrieves another result: those with higher contrast (*Leona Blanca* and *Chola*) have lower *MC* values. This might be attributed to the occurrence of lower polarity surface composition regions in these two types and a predominant higher polarity surface (without contrast) in the other two kinds. *TSM* measurements agree with this analysis when comparing starch sources of different varieties.

Mechanical tests performed showed that as the percentage of glycerol increases, the tensile strength decreases, and elongation at break increases. This could correlate to the topographical conformations observed at the nanoscale, since films generated from starch from all varieties exhibit a predominantly granular structure without glycerol, which diminishes to some extent when plasticizer concentration is increased.

## 4. Conclusions

This study involved the analysis of films cast from starch extracted from four different Ecuadorian potato varieties (*Leona Blanca, Única, Chola,* and *Santa Rosa*) blended with glycerol at different concentrations (0, 1, 2, 3, and 4% *w*/*v*). It was observed that potato starch-based films exhibit varying properties depending on the starch source. Macroscopic properties, such as elastic properties, visible optical absorption, water vapor permeability, moisture content, and solubility, were compared to the nanoscopic topography and phase contrast data recorded by using atomic force microscopy. It was found in most cases that, without glycerol, films present a grainy structure with reduced tensile deformation at break. The addition of glycerol flattens their surface at low concentrations but produces smooth-sided microscopic protrusions at higher concentrations. At the same time, increasing this plasticizer’s concentration apparently induces a decrease in the tensile strength and an elongation at break enhancement. Rising the amount of glycerol incorporated growths the water vapor permeability, the moisture content, and the total soluble matter of the corresponding films.

Starch films extracted from the *Chola* potato variety behave slightly dissimilarly. They demonstrated a higher potentiality for resistant film formation, exhibiting an increased strength to rupture, lower solubility, and reduced moisture content, more adequate for a rigid containers’ use. These characteristics are consistent with the topography and phase-contrast analyses made at the nanoscale.

Appropriate starch source selection would depend on the specific applicability required. If more plastic deformation is needed, *Única* is probably a better choice. If, instead, solubility is demanded, the *Santa Rosa* variety could be chosen. If, on the other hand, lower solubility is sought because of the ambient humidity, *Leona Blanca* without glycerol may be more suitable. Although all potato species contain starch capable of generating biofilms using easy and cheap extraction protocols, significant property differences were found among them. The optimal potato type that should be selected in each case would depend on the particular use the biofilms were demanded for.

## Figures and Tables

**Table 1:** Absorbance measured iodine solutions and determined amylose molar concentrations of starch extracted from each potato variety.

Potato Variety	Absorption (A.U.)	Weight-Weight Concentration (%)
Leona Blanca	0.309 ± 0.008	23.8 ± 0.6 ^a^
Única	0.273 ± 0.006	21.0 ± 0.5 ^b^
Chola	0.335 ± 0.014	25.8 ± 1.0 ^a^
Santa Rosa	0.256 ± 0.014	19.7 ± 1.0 ^b^

Different letters (a, b) in the same column indicate significant differences between the different films (*p* ≤ 0.05).

**Table 2:** Thickness (mm) of each biofilm formed from starch extracted from different potato varieties.

% *w*/*v* Glycerol	*Leona Blanca*	*Única*	*Chola*	*Santa Rosa*
0	0.42 ± 0.02 ^a^	0.20 ± 0.01 ^b^	0.14 ± 0.01 ^d^	0.18 ± 0.02 ^c^
1	0.28 ± 0.01 ^b^	0.13 ± 0.01 ^c^	0.11 ± 0.01 ^d^	0.38 ± 0.02 ^a^
2	0.24 ± 0.01 ^a^	0.21 ± 0.01 ^c^	0.22 ± 0.01 ^b^	0.21 ± 0.01 ^c^
3	0.21 ± 0.01 ^d^	0.22 ± 0.01 ^c^	0.29 ± 0.02 ^a^	0.27 ± 0.01 ^b^
4	0.38 ± 0.02 ^a^	0.27 ± 0.01 ^c^	0.32 ± 0.01 ^b^	0.28 ± 0.01 ^c^

Different letters (a, b, c, d) in the same row indicate significant differences between the different biofilms (*p* ≤ 0.05).

**Table 3:** Opacity values (AU/mm) obtained from each of the biofilms formed from starch from different potato varieties and glycerol percentages.

% *w*/*v* Glycerol	*Leona Blanca*	*Única*	*Chola*	*Santa Rosa*
0	0.45 ± 0.13 ^b^	0.41 ± 0.10 ^b^	1.12 ± 0.15 ^a^	0.44 ± 0.10 ^b^
1	1.01 ± 0.10 ^b^	0.51 ± 0.10 ^bc^	1.94 ± 0.40 ^a^	0.40 ± 0.15 ^c^
2	1.43 ± 0.21 ^a^	0.55 ± 0.10 ^b^	1.40 ± 0.32 ^a^	0.68 ± 0.10 ^b^
3	1.15 ± 0.15 ^a^	0.46 ± 0.10 ^c^	0.78 ± 0.10 ^b^	0.52 ± 0.10 ^c^
4	1.68 ± 0.32 ^a^	0.60 ± 0.11 ^c^	1.66 ± 0.68 ^a^	0.73 ± 0.12 ^b^

Different letters (a, b, c) in the same row indicate significant differences between the different biofilms (*p* ≤ 0.05).

**Table 4:** Water vapor permeability (*WVP*) data (mg mm min^−1^ m^−2^ kPa^−1^) obtained from each of the biofilms formed from starch from different potato varieties and glycerol percentages.

% *w*/*v* Glycerol	*Leona Blanca*	*Única*	*Chola*	*Santa Rosa*
0	24 ± 4 ^a^	20 ± 5 ^a^	16 ± 5 ^a^	13 ± 5 ^a^
1	33 ± 6 ^a^	24 ± 7 ^a^	13 ± 5 ^a^	22 ± 9 ^a^
2	34 ± 2 ^a^	34 ± 11 ^a^	19 ± 8 ^a^	27 ± 8 ^a^
3	39 ± 13 ^a^	41 ± 7 ^a^	28 ± 5 ^a^	30 ± 3 ^a^
4	45 ± 12 ^a^	43 ± 6 ^a^	29 ± 5 ^a^	28 ± 6 ^a^

Letter a in the same row indicates no significant difference among the biofilms obtained from the types analyzed (*p* > 0.05).

**Table 5:** Moisture content (*MC*) data obtained from each of the biofilms formed from starch from different potato varieties and glycerol percentages.

% *w*/*v* Glycerol	*Leona Blanca*	*Única*	*Chola*	*Santa Rosa*
0	12.86 ± 0.64 ^c^	27.16 ± 0.19 ^a^	11.97 ± 0.38 ^c^	25.76 ± 0.68 ^b^
1	26.61 ± 0.73 ^b^	25.71 ± 0.29 ^b^	20.42 ± 0.74 ^c^	31.12 ± 0.74 ^a^
2	32.97 ± 0.82 ^b^	37.74 ± 0.96 ^a^	32.13 ± 0.70 ^b^	36.04 ± 0.29 ^a^
3	43.83 ± 0.71 ^b^	46.09 ± 0.69 ^a^	37.42 ± 0.66 ^c^	38.61 ± 0.50 ^c^
4	52.13 ± 0.72 ^a^	49.83 ± 0.16 ^b^	45.42 ± 0.52 ^c^	44.77 ± 0.75 ^c^

Different letters (a, b, c) in the same row indicate significant differences among distinct biofilms (*p* ≤ 0.05).

**Table 6:** Total Soluble Matter in water (*TSM*) data obtained from each of the biofilms formed from starch from different potato varieties and glycerol percentages.

% *w*/*v* Glycerol	*Leona Blanca*	*Única*	*Chola*	*Santa Rosa*
0	19.09 ± 0.73 ^d^	38.94 ± 0.14 ^b^	29.23 ± 0.74 ^c^	66.40 ± 0.47 ^a^
1	23.49 ± 0.10 ^c^	25.52 ± 0.90 ^b^	22.74 ± 0.95 ^c^	52.84 ± 0.79 ^a^
2	35.35 ± 0.85 ^c^	38.40 ± 0.97 ^b^	34.83 ± 0.78 ^c^	58.55 ± 0.74 ^a^
3	39.11 ± 0.61 ^c^	46.50 ± 0.92 ^b^	45.53 ± 0.95 ^b^	65.43 ± 0.83 ^a^
4	52.52 ± 0.76 ^b^	52.51 ± 0.83 ^b^	50.89 ± 0.88 ^b^	65.39 ± 0.56 ^a^

Different letters (a, b, c, d) in the same row indicate significant differences among distinct types (*p* ≤ 0.05).

## Data Availability

Data are contained within the article and Supplementary Materials.

## Supplementary Materials

The following supporting information can be downloaded at https://www.mdpi.com/article/10.3390/polym16131873/s1, Figure S1: Optical reflection at white light; Table S1, Table S2, Table S3: Roughness analysis of the AFM images; Figure S2: 3D topographic AFM images; Figure S3: Phase contrast AFM images.
