# Supplementary material for: Nanoscopic Characterization of Starch-Based Biofilms Extracted from Ecuadorian Potato (Solanum tuberosum) Varieties
Source: Polymers (Basel). 2024 Jun 30;16(13):1873. doi: 10.3390/polym16131873 (PMC11244412; doi:10.3390/polym16131873)
Supplement: Supplementary file 1 [file polymers-16-01873-s001.zip › polymers-3031769-supplementary.pdf]

Supplementary

Nanoscopic Characterization of Starch-based Biofilms Extracted from Ecuadorian potato (*Solanum tuberosum*) varieties

Pablo Ilvis [1], José Acosta [1], Mirari Arancibia [1,*], and Santiago Casado [1,*]

[1] Food and Biotechnology Science and Engineering Department, Technical University of Ambato, Ambato 180207, Ecuador
* Correspondence: marancibias@uta.edu.ec; s.casado@uta.edu.ec

Supplementary Materials:

S.1. Optical reflection at white light

Morphologies observed in white light reflective optical microscopy.

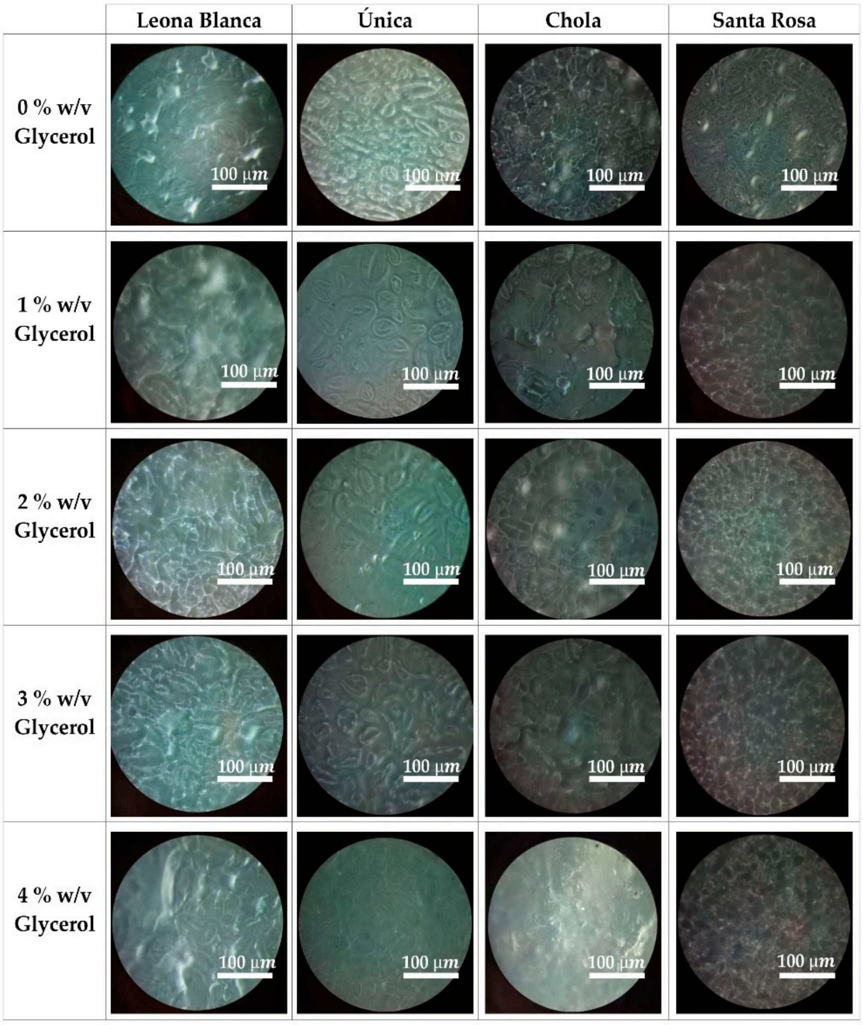

Figure S1. Micrographs of the starch biofilm samples from different varieties of starch and glycerol percentages obtained through the optical white light reflection microscope.

S.2. Roughness analysis of the AFM images

Table S1. Ra roughness values (μm) of the AFM topography images of the biofilms based on starch extracted from each potato variety blended with different glycerol percentages, shown in Figure 5. This magnitude measures the average roughness between the profile and its mean surface.

% w/v Glycerol	*Leona Blanca*	*Única*	*Chola*	*Santa Rosa*
0	0.0151	0.0218	0.0094	0.0288
1	0.0048	0.0040	0.0101	0.0144
2	0.0125	0.0124	0.0131	0.0290
3	0.0402	0.0173	0.0283	0.0297
4	0.0425	0.0170	0.0755	0.0384

Table S2. Rz roughness values (μm) of the AFM topography images of the biofilms based on starch extracted from each potato variety blended with different glycerol percentages, shown in Figure 5. This magnitude measures the arithmetic average of the five highest peaks and five lowest valleys.

% w/v Glycerol	*Leona Blanca*	*Única*	*Chola*	*Santa Rosa*
0	0.1427	0.1731	0.0859	0.2570
1	0.0439	0.0387	0.0975	0.1541
2	0.1219	0.1177	0.1070	0.2280
3	0.3336	0.1224	0.2570	0.2479
4	0.3972	0.1493	0.4716	0.2681

Table S3. Rq roughness values (μm) of the AFM topography images of the biofilms based on starch extracted from each potato variety blended with different glycerol percentages, shown in Figure 5. This magnitude measures the root mean squared roughness.

% w/v Glycerol	*Leona Blanca*	*Única*	*Chola*	*Santa Rosa*
0	0.0190	0.0272	0.0122	0.0372
1	0.0060	0.0050	0.0132	0.0185
2	0.0159	0.0156	0.0162	0.0361
3	0.0495	0.0176	0.0378	0.0373
4	0.0531	0.0215	0.0919	0.0484

S.3. 3D topographic AFM images

Tridimensional surface topographic of each analyzed biofilm measured using the Atomic Force Microscopy (AFM).

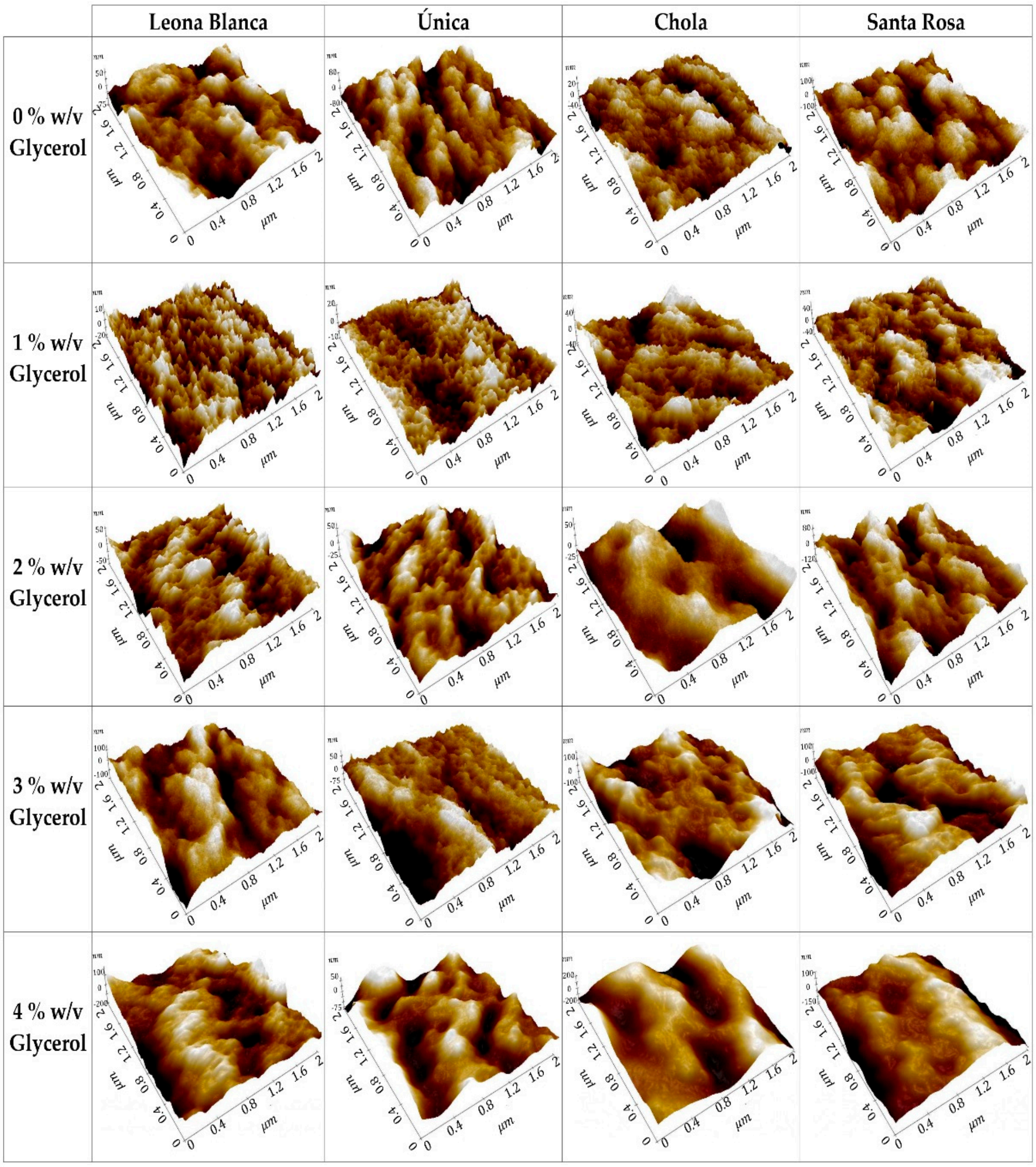

Figure S2. Tridimensional topographic images from Atomic Force Microscopy obtained on the surface of biofilms based on starch extracted from each potato variety blended with different glycerol percentages.

S.4. Phase contrast AFM images

Surface phase contrast of each analyzed biofilm measured using the Atomic Force Microscopy (AFM).

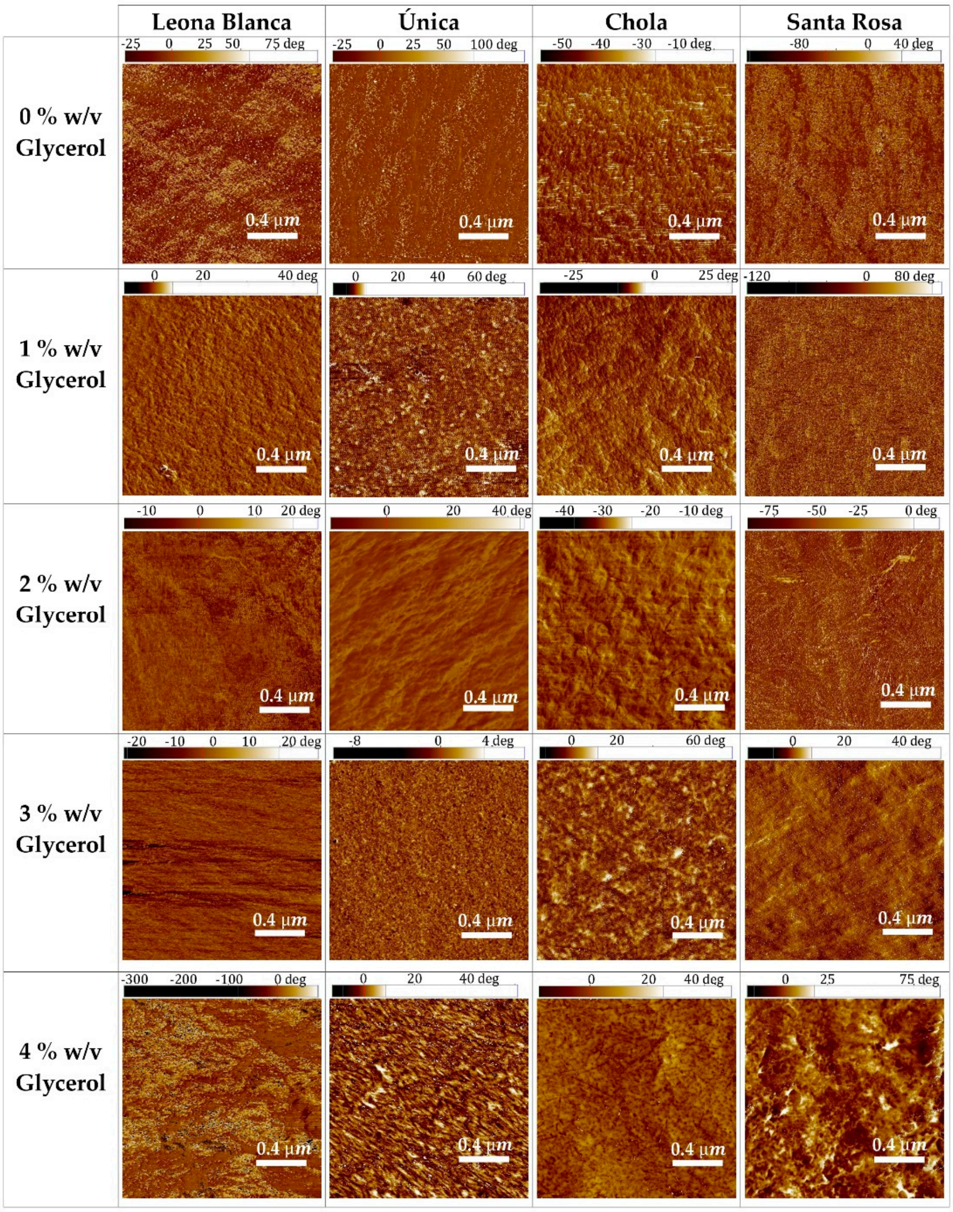

Figure S3. Phase contrast images from Atomic Force Microscopy obtained in tapping mode on the surface of biofilms based on starch extracted from each potato variety blended with different glycerol percentages.
